# Crystal structure and Hirshfeld surface analysis of *cyclo*-tetra­bromido-1κ^2^ *Br*,3κ^2^ *Br*-tetra­kis­(μ_2_-2-{[(pyridin-2-yl)meth­yl]amino}­ethane-1-thiol­ato-κ^3^ *N*,*S*:*S*)tetra­mercury(II)

**DOI:** 10.1107/S205698902300823X

**Published:** 2023-09-29

**Authors:** Isla D. Thomas, Kathryn R. Kocher, Julie A. Viehweg, Robert D. Pike, Deborah C. Bebout

**Affiliations:** a Department of Chemistry, William & Mary, Williamsburg, VA 23187-8795, USA; Universität Greifswald, Germany

**Keywords:** crystal structure, Hirshfeld surface analysis, metallacycle, chelating *N*,*S*-ligands, Hg^2+^ complex

## Abstract

The macrometallacyclic title compound, [Hg_4_Br_4_(C_8_H_11_N_2_S)_4_] or [((Hg**L**_2_)(HgBr_2_))_2_] (**1**) where **HL** = 2-{[(pyridin-2-yl)meth­yl]amino}­ethane-1-thiol, was prepared and structurally characterized. The Hg^2+^ complex crystallizes in the *P*2_1_/*c* space group. The centrosymmetric Hg_4_S_4_ metallacycle is constructed from metal ions with alternating distorted tetra­hedral Br_2_S_2_ and distorted seesaw N_2_S_2_ primary coordination environments with pendant pyridyl groups. The backfolded extended chair metallacycle conformation suggests inter­actions between each of the bis-chelated mercury atoms and Br atoms lying above and below the central Hg_2_S_4_ plane. Supra­molecular inter­actions in **1** include a fourfold aryl embrace and potential hydrogen bonds with bromine as the acceptor. Hirshfeld surface analysis indicates that H⋯H (51.7%), Br⋯H/H⋯Br (23.0%) and C⋯H/H⋯C (9.5%) inter­actions are dominant.

## Abstract 2

A macrometallacyclic mercury(II) complex [((Hg**L**_2_)(HgBr_2_))_2_], where **HL** = 2-{[(pyridin-2-yl)meth­yl]amino}­ethane-1-thiol, was synthesized and characterized by single-crystal X-ray diffraction and Hirshfeld analysis.

## 1. Chemical context

For many years, we and others have been inter­ested in the structures of group 12 coordination compounds including an amino­ethane­thiol­ate moiety (Hu *et al.*, 2020 ▸; Hallinger *et al.*, 2017 ▸; Akhtar *et al.*, 2015 ▸; Bharara *et al.*, 2006*a* ▸,*b* ▸; Fleischer *et al.*, 2006 ▸; Viehweg *et al.*, 2010 ▸; Brand & Vahrenkamp, 1995 ▸; Avdeef *et al.*, 1992 ▸; Tuntulani *et al.*, 1992 ▸; Kaptein *et al.*, 1987 ▸). Single-crystal X-ray diffraction is critical to the characterization of group 12 amino­ethane­thiol­ate complexes since the nuclearity of complexes with 1:1 metal-to-ligand ratio can vary with the identity of ancillary ligands and counter-ions (Brennan *et al.*, 2022 ▸; Lai *et al.*, 2013 ▸; Brand & Vahrenkamp, 1995 ▸) and complexes with novel ring structures have been produced (Ritz *et al.*, 2019 ▸; Viehweg *et al.*, 2010 ▸). 
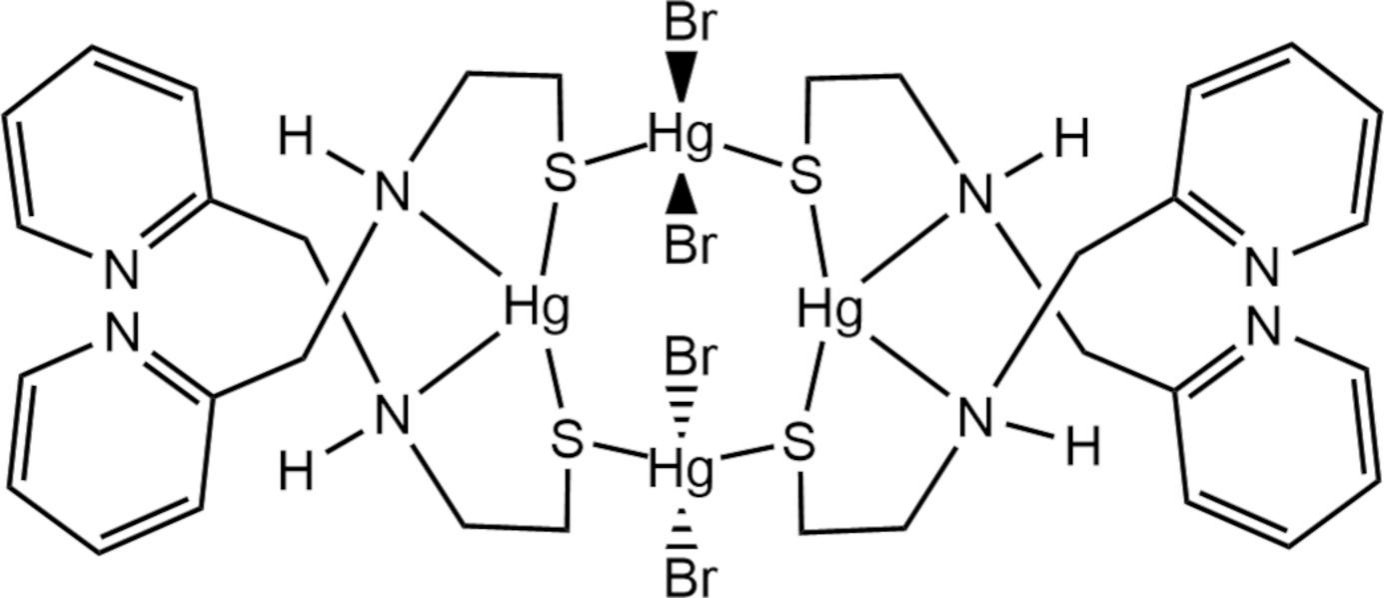


In contrast to the polymeric [Zn**L** *X*]_*n*_ structure reported for zinc halide complexes of deprotonated **HL** = 2-{[(pyridin-2­yl)meth­yl]amino}­ethane-1-thiol (Brand & Vahrenkamp, 1995 ▸), the cyclic tetra­nuclear compound *cyclo*-tetra­bromido-1κ^2^ *Br*,3κ^2^ *Br*-tetra­kis­(μ_2_-2-{[(pyridin-2-yl)meth­yl]amino}­ethane-1-thiol­ato-κ^3^ *N*,*S*:*S*)tetra­mercury(II) (**1**) constructed from alternating Hg**L**­_2_ and HgBr_2_ centers was found for the mercuric bromide complex of **L**. This communication reports the preparation, crystal structure, and Hirshfeld surface analysis of **1**, which facilitate an in-depth discussion of its structural features.

## 2. Structural commentary

**Figure 1:**
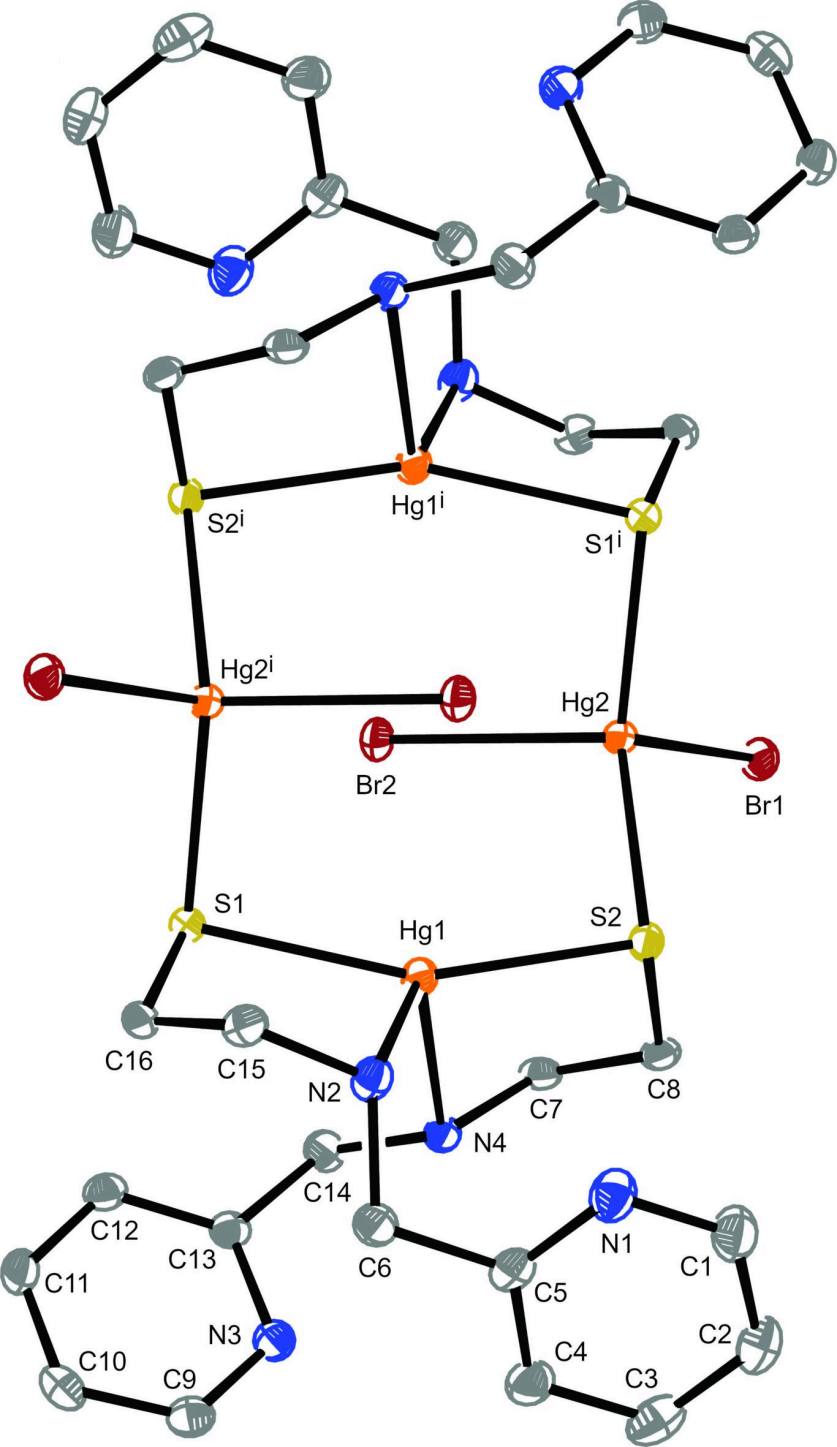
The mol­ecular structure of **1** with the atom numbering scheme generated with *ORTEP-3 for Windows* (Farrugia, 2012 ▸). Displacement ellipsoids are drawn at the 50% probability level. Symmetry code: (i) −*x* + 1, −*y* + 1, −*z* + 1.

Complex **1** crystallizes as discrete centrosymmetric mol­ecules with an eight-membered metallacycle of alternating mercury and sulfur atoms (Fig. 1 ▸). The asymmetric unit contains Hg_2_ **L**_2_Br_2_, which is one half of complex **1**. The sets of four mercury(II) centers and four sulfur atoms in **1** each lie rigorously in their own plane, as required by the crystallographic inversion center located in the center of the mol­ecule. The angle between these planes is 25.741 (18)°. The two Hg2 atoms are approximately tetra­hedrally coordinated to two terminal bromines and two bridging sulfur atoms (Table 1 ▸). These are separated by bis-chelated Hg1 metal atoms. The potentially tridentate ligand has an N-μ_2_-S coordination mode with a pendant pyridyl ring. The pyridyl nitro­gen atoms are located 3.563 (4)–4.303 (3) Å from the closest mercury atoms and oriented unfavorably for either intra- or inter­molecular bonding inter­actions with a metal center. The chelate rings have an envelope conformation with the methyl­ene carbon in the flap position. The Hg1 metal atoms show a marked distortion from tetra­hedral towards seesaw coordination with a widened S—Hg—S angle of 156.40 (3)°. The two bridging Hg—S distances are slightly longer and more similar (Δ = 0.003 Å) than the two chelating Hg–S distances (Δ = 0.030 Å).

Alternatively, the Hg_4_S_4_ ring can be viewed as an extended chair containing a central planar Hg_2_S_4_ arrangement with one backfolded mercury atom on each side of the plane. The six Hg_2_S_4_ atoms lie between 0.0653 (3) and 0.1079 (5) Å from the mean plane. The HgS_2_ planes forming the head and foot of the chair are in an unusually acute 83.99 (4)° angle with the central plane placing Hg2 and Br2 over the Hg1_2_S_4_ plane. Furthermore, the Hg2—Br2 bonds are 0.084 Å longer than the Hg2—Br1 bonds. These observations imply some weak inter­actions between the Br2 atoms and the two bis-chelated mercury atoms located 3.3951 (5) Å and 3.6026 (5) Å away. In a similar setting, such likely inter­actions between group 12 metal ions and halides have been reported for complexes of 2-(di­methyl­amino)­ethane­thiol­ate (Casals *et al.*, 1991 ▸). Further­more, a related penta­cyclic Cd_4_S_4_Cl_2_ primary bonding core has been reported for [(Cd(SC(CH_3_)_2_CH_2_NH_2_)_2_(CdCl_2_)]_2_·2H_2_O (refcode MEASCD; Fawcett *et al.*, 1978 ▸). The Hg⋯Hg separation between the bis-brominated mercury atoms [5.0530 (6) Å] is shorter than the distance between the bis-chelated mercury atoms [5.4023 (5) Å], both of which are too long for significant inter­actions between the metal atoms. In contrast, [Cu**L**]_4_ has mono-N,S chelated metal atoms in a *D*_2*d*_ butterfly arrangement with Cu⋯Cu separations of 2.6957 (11) and 3.370 (1) Å (refcode TEVMAI; Stange *et al.*, 1996 ▸).

## 3. Supra­molecular features

**Figure 2:**
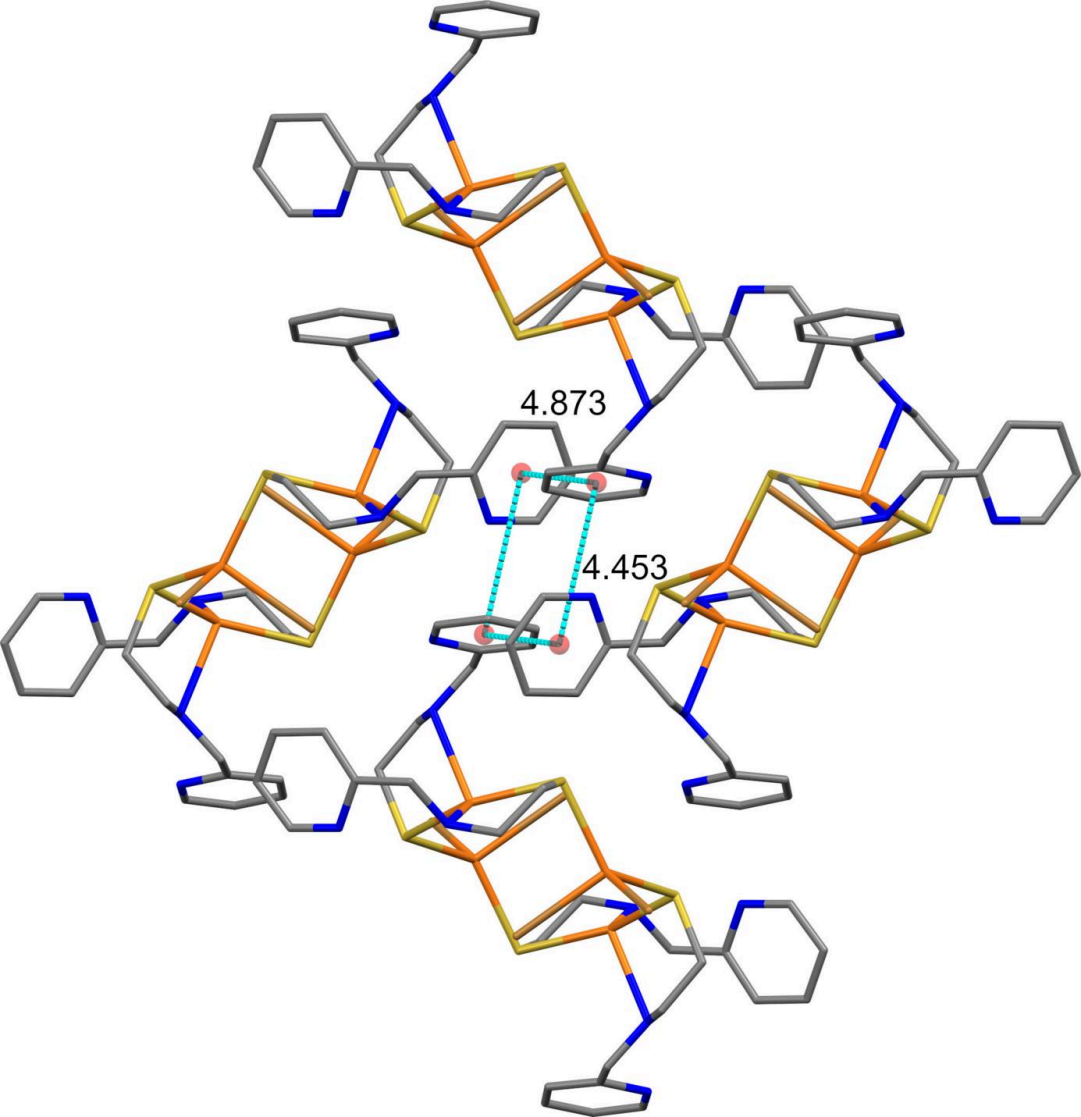
Fourfold aryl embrace between two pairs of inversion-related mol­ecules of **1** viewed down the *a* axis illustrated using *Mercury* (Macrae *et al.*, 2020 ▸). Hydrogen atoms were omitted for clarity. Ring centroids are shown as red spheres. Cyan dashed lines show centroid–centroid distances (for additional numerical data, see Table 2 ▸).

**Figure 3:**
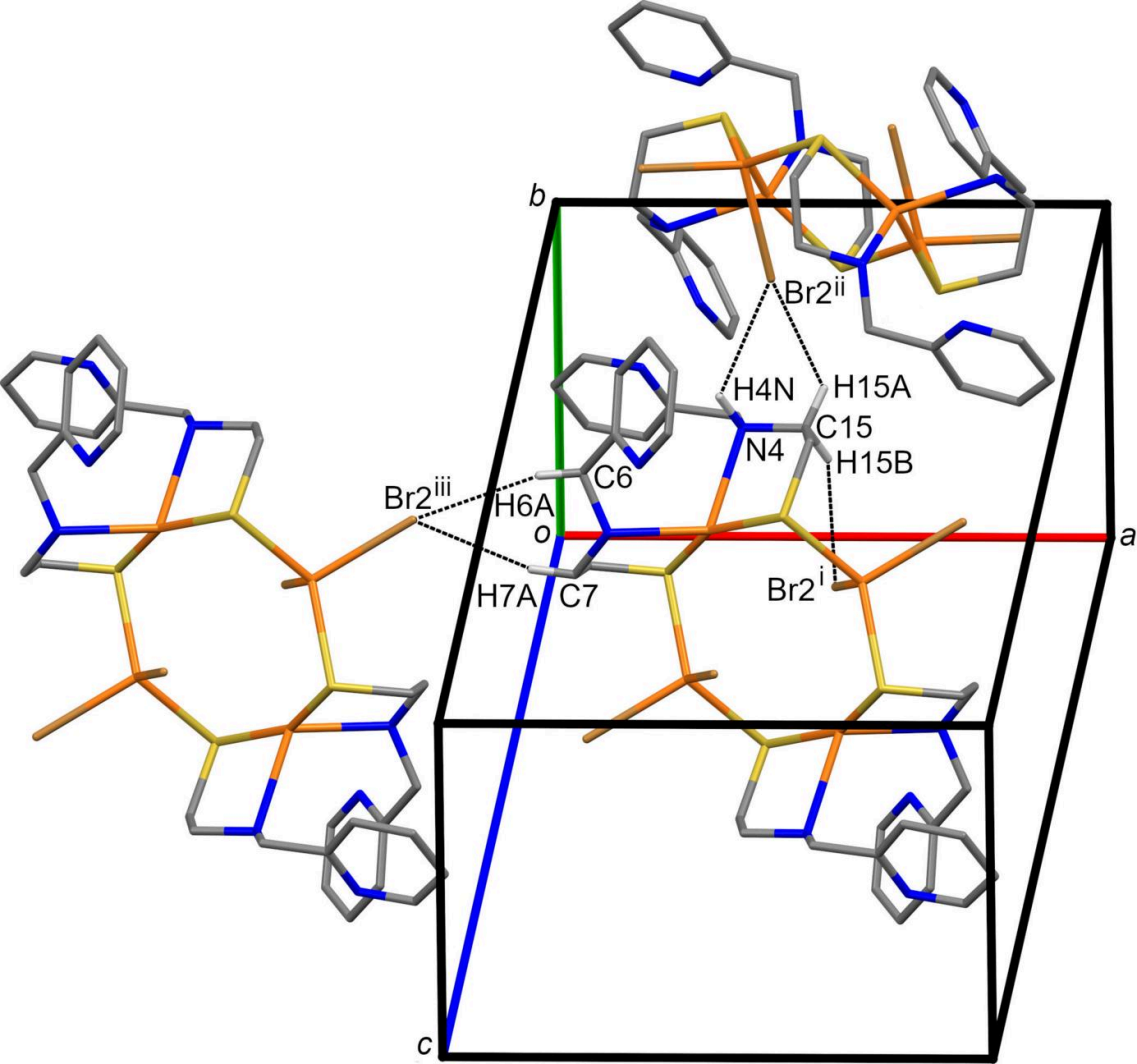
A view of N—H⋯Br and C—H⋯Br hydrogen bonds in compound **1** shown as dashed lines illustrated using *Mercury* (Macrae *et al.*, 2020 ▸). Symmetry codes as in Table 3 ▸.

In addition to a variety of van der Waals contacts, the packing of **1** is stabilized by π–π inter­actions (Fig. 2 ▸ and Table 2 ▸) and hydrogen bonding (Fig. 3 ▸ and Table 3 ▸). The pendant pyridyl rings (centroids *Cg*1: N1/C1–C5; *Cg*2: N3/C9–C13) participate in a fourfold aryl embrace around a crystallographic inversion center with centroid–centroid distances of 4.453 (2) and 4.873 (2) Å (Table 2 ▸). The pyridyl planes subtend an angle of 62.87 (13)°. Most of the hydrogen bonds involve C—H donors and Br acceptors (Brammer *et al.*, 2001 ▸). Neither nitro­gen atom of the pendant pyridyl rings participates in inter­molecular hydrogen bonding.

## 4. Hirshfeld surface analysis

**Figure 4:**
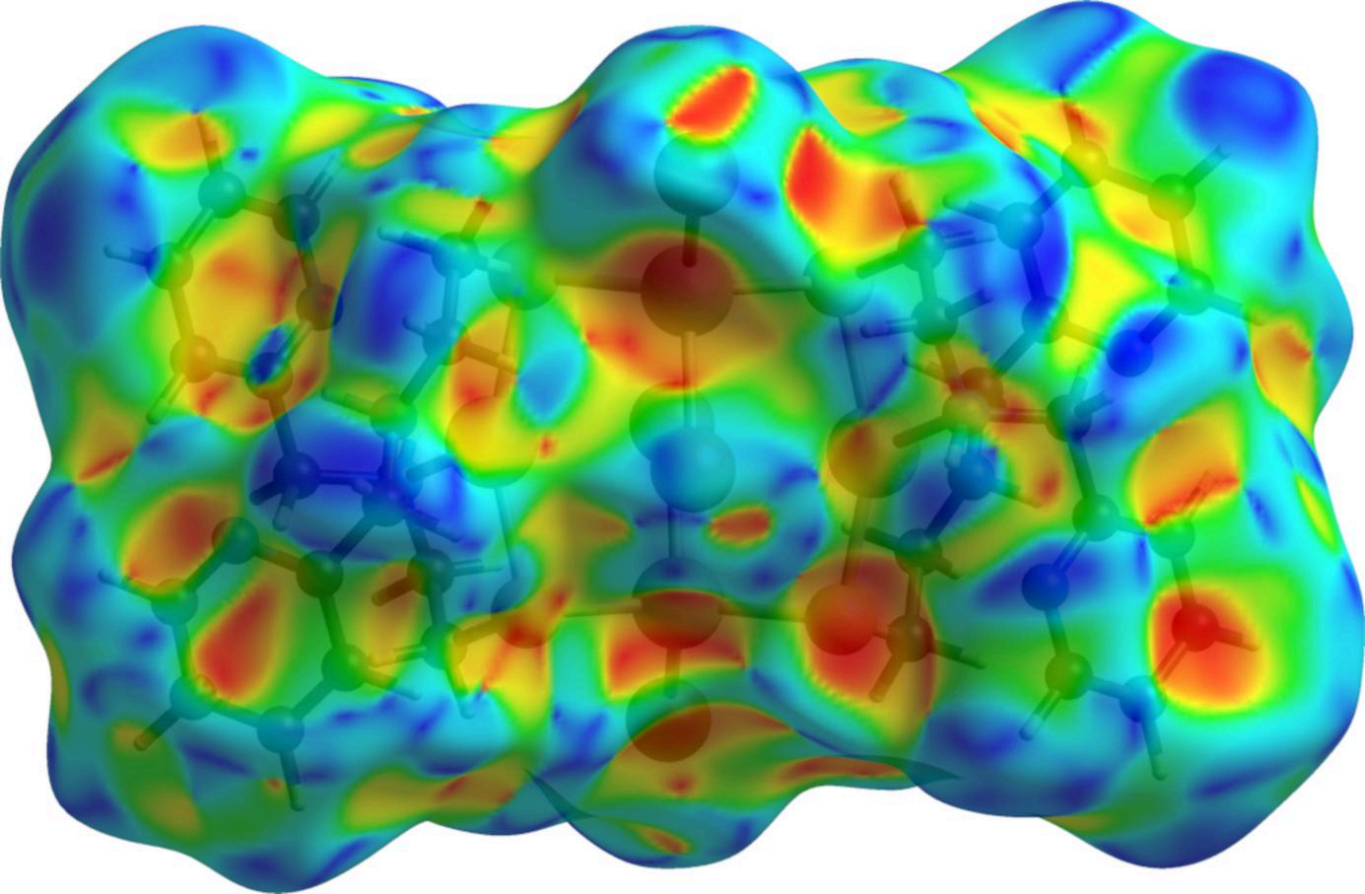
Hirshfeld surface of **1** plotted over shape-index generated with *CrystalExplorer21.5* (Spackman *et al.*, 2021 ▸). Blue and red areas represent bumps and hollow regions, respectively, on the shape-index surface.

Inter­molecular inter­actions were investigated by qu­anti­tative analysis of the Hirshfeld surface and visualized with *CrystalExplorer 21.5* (Spackman *et al.*, 2021 ▸). The Hirshfeld surface of **1** plotted over shape-index did not have the hourglass figures associated with face-to-face aromatic inter­actions (Fig. 4 ▸). Instead, circular and wedge-shaped blue bumps associated with N3 as the edge of the pyridyl ring (top right) complement a pair of similarly shaped red pits on the face of the N1 pyridyl ring (lower right). The reverse side of the N1 pyridyl ring (upper left) has a multicolored iris-like feature and a large blue bump while complementary characteristics lie across the center of the mol­ecule. A feature reminiscent of a paw print with red digital pad pits extending over the reverse face of the N3 pyridyl ring and a blue metacarpal pad bump over the attached methyl­ene (lower left) complements a reverse-colored paw print overlying the inner edge of the N1 pyridyl ring.

**Figure 5:**
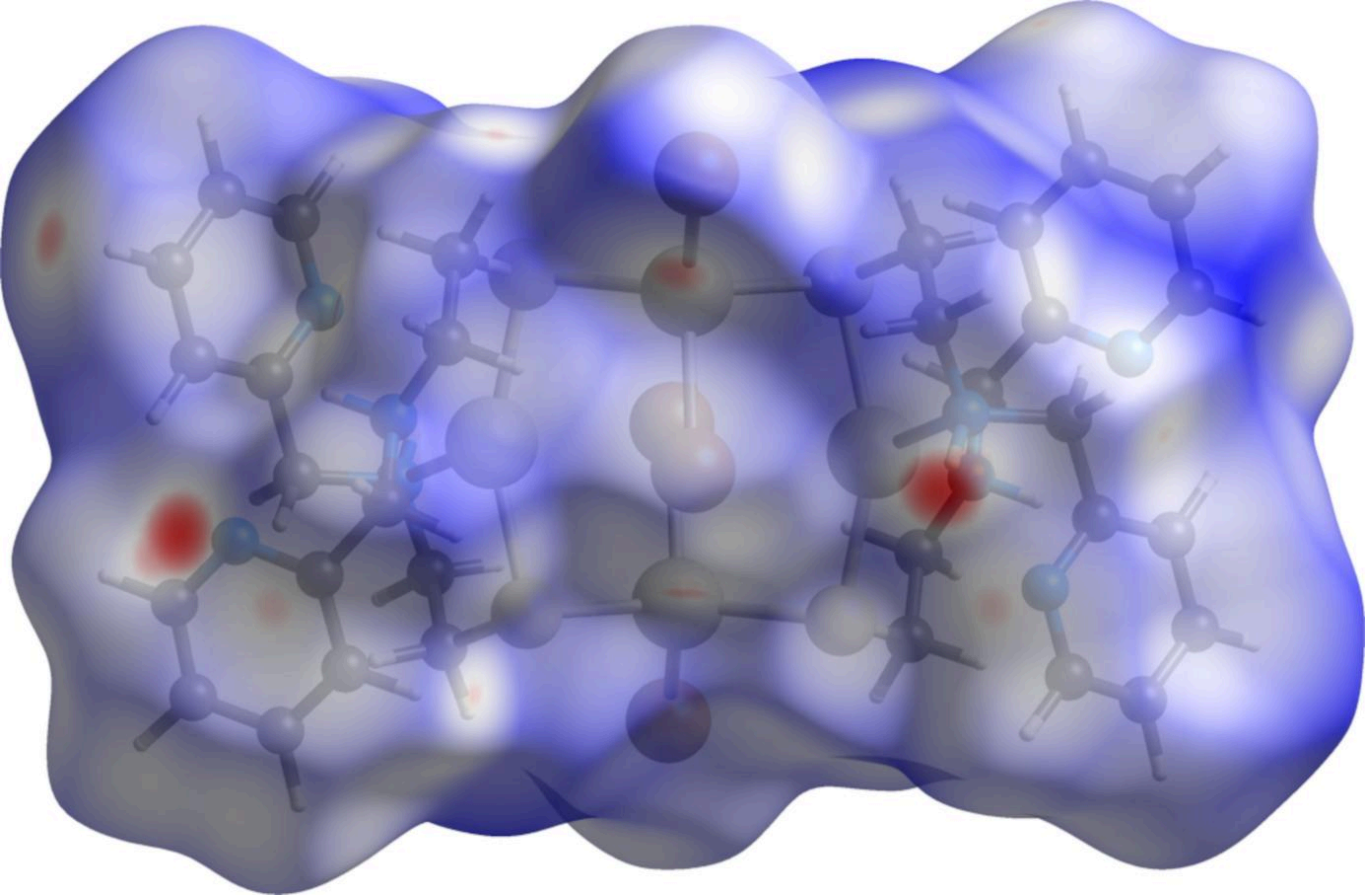
Hirshfeld surface of **1** plotted over normalized contact distance (*d*_norm_) in the range from −0.2689 (red) to 1.5908 (blue) a.u. generated with *CrystalExplorer21.5* (Spackman *et al.*, 2021 ▸).

The Hirshfeld surface of **1** mapped with the function *d*_norm_, the sum of the distances from a surface point to the nearest inter­ior (*d*_i_) and exterior (*d*_e_) atoms normalized by the van der Waals (vdW) radii of the corresponding atom (rvdW), is shown in Fig. 5 ▸. Contacts near and longer than the sum of van der Waals radii are shown in white and blue, respectively. Red areas are observed for atoms associated with close contacts at least 0.050 Å shorter than the sum of van der Waals radii (Bondi, 1964 ▸). The most intense red spots correspond to an inter­molecular contact between H2*N* and adjacent atoms N3 and C9. Neighboring pale-red regions reflect a contact between N1 and C13, respectively. Medium intensity red spots are associated with Br1 and H3.

**Figure 6:**
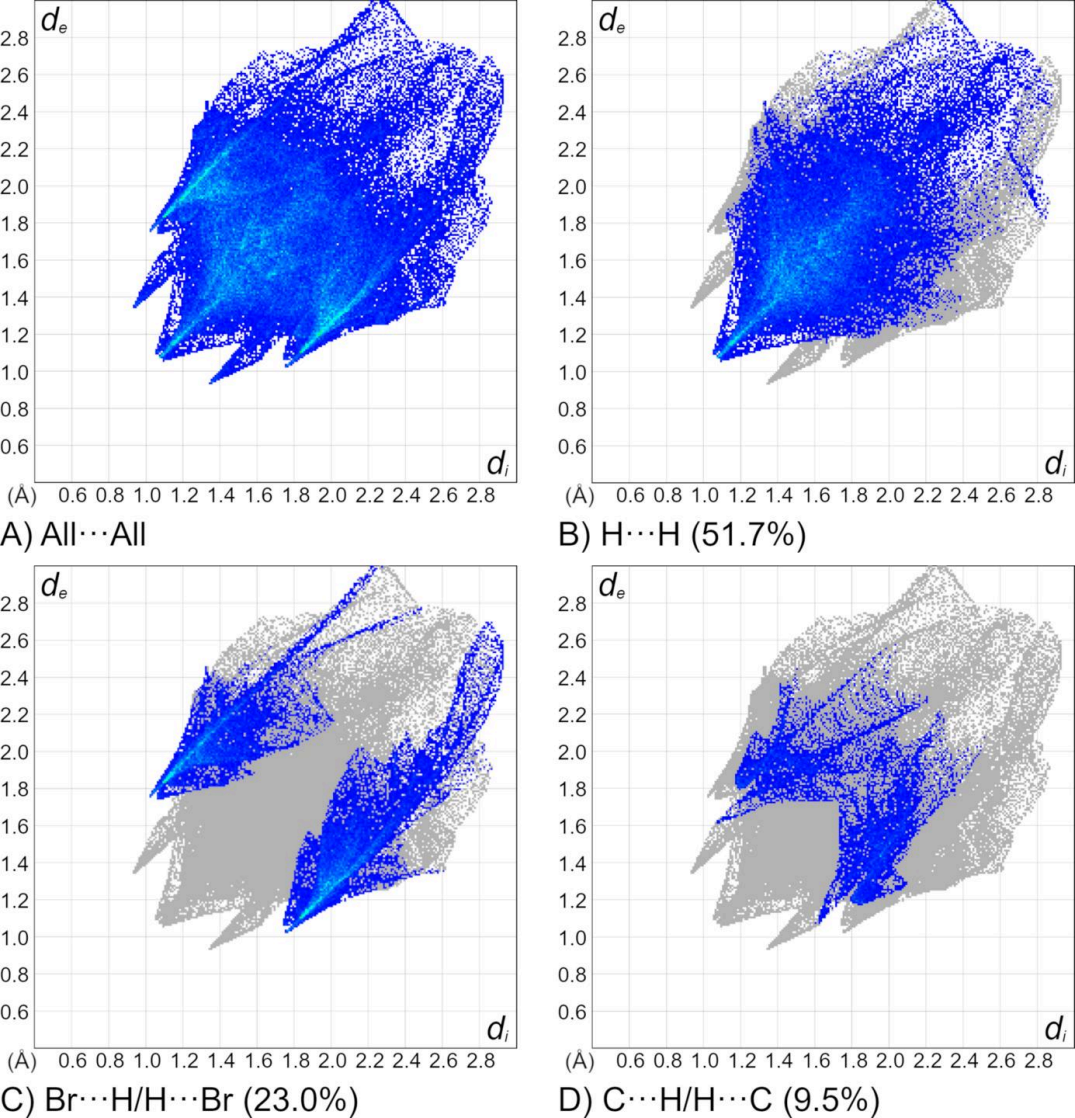
The full two-dimensional fingerprint plots for **1**, showing (*a*) all inter­actions, and components delineated into (*b*) H⋯H, (*c*) Br⋯H/H⋯Br and (*d*) C⋯H/H⋯C inter­actions generated with *CrystalExplorer* 21.5 (Spackman *et al.*, 2021 ▸). The *d*_i_ and *d*_e_ values are closest inter­nal and external distances (in Å) from given points on the Hirshfeld surface.

The overall 2D fingerprint plot for **1** is provided in Fig. 6 ▸ *a* while the inter­actions delineated into H⋯H (51.7%), Br⋯H/H⋯Br (23.0%), and C⋯H/H⋯C (9.5%) contacts are shown in Fig. 6 ▸ *b*–*d*. Other minor contributions to the Hirshfeld surface are from S⋯H/H⋯S (7.5%), N⋯H/H⋯N (4.7%), N⋯C/C⋯N (1.8%), Hg⋯H/H⋯Hg (1.4%), Br⋯C/C⋯Br (0.3%), N⋯N (0.1%), and C⋯C (0.1%) inter­actions.

## 5. Database survey

A search of the Cambridge Structural Database (CSD, Version 5.44, update of April 2023; Groom *et al.* 2016 ▸) using *ConQuest* (Bruno *et al.*, 2002 ▸) for metal complexes of **L** yielded 21 hits. Most of the complexes feature an *N,N′-μ_2_*-*S* binding mode for **L** including [Pd**L**]_4_Cl(ClO_4_)_3_·CH_3_OH·H_2_O (refcode SUZDUM; Kawahashi *et al.*, 2001 ▸), which had a Pd_4_S_4_ metallocycle with boat conformation. Salts of group 12 metal ions with weakly coordinating perchlorate and tetra­fluoro­borate counter-ions have generated a variety of solvated complex ions with composition [Zn_3_ **L**_4_]^2+^ (refcode BITNIB: Mikuriya *et al.*, 1998 ▸; JEHWEB: Hallinger *et al.*, 2017 ▸; ZACWAB; Brand & Vahrenkamp, 1995 ▸) and [HgZn_2_ **L**_4_]^2+^ (refcodes JEHWIF, JEHWOL, JEHWUR, JEHXAY, JEHXEC; Hallinger *et al.*, 2017 ▸). Additional complexes of tridentate **L** with group 12 metal ions include [Hg_5_ **L**_6_](ClO_4_)_4_·toluene (DABJIB; Viehweg *et al.*, 2010 ▸), [Zn**L**Cl]_n_ (ZACWEF; Brand & Vahrenkamp, 1995 ▸), [Zn**L**(acetato-*O*)]_2_ (ZACWIJ; Brand & Vahrenkamp, 1995 ▸), and [Zn**L**(quinoline-2-carboxyl­ato-*N*,*O*)] (ZACWUV; Brand & Vahrenkamp, 1995 ▸). The only complexes of **L** with pendant pyridyl rings are [Mo**L**(S_2_)_2_O] (refcode OTUHER; Wei *et al.*, 2011 ▸), [Zn**L**_2_] (refcode ZACWOP; Brand & Vahrenkamp, 1995 ▸) and [Cu**L**]_4_ (refcode TEVMAI; Stange *et al.*, 1996 ▸).

A search of the Cambridge Structural Database (CSD, Version 5.44, update of April 2023; (Groom *et al.*, 2016 ▸) using *ConQuest* (Bruno *et al.*, 2002 ▸) for Hg_4_S_4_ metallacycles yielded eight tetra­mercury hits. Complexes with chelating N and S donor amino­ethane­thiol­ate ligands share an extended chair conformation comparable to **1** with varying degrees of backfolding (refcode IKUVUH: Clegg, 2016 ▸; refcodes DENKUD and DENLEO: Bharara *et al.*, 2006*a* ▸; refcode ECIYOF: Bharara *et al.*, 2006*b* ▸; refcode JIZWEU: Casals *et al.*, 1991 ▸). Complexes with separate pyridyl and alkyl­thiol­ate ligands exhibit nearly planar Hg_4_S_4_ rings cinched across the middle by a pair of μ-Cl ligands (refcode BTCHGP: Canty, *et al.*, 1978 ▸; refcode TBTPHG: Canty, *et al.*, 1979 ▸). In contrast, a chair ring conformation with only four coplanar atoms and μ-Cl was observed with di­propyl­dithio­carbamate ligands (XOKPAR: Loseva *et al.*, 2019 ▸).

## 6. Synthesis and crystallization

A solution of HgBr_2_ (942 mg, 2.61 mmol) in 15 mL methanol was added to a stirred solution of **LH** (445 mg, 2.64 mmol) and NaOH (104 mg, 2.60 mmol) in 20 mL methanol. A white precipitate characterized as **1** was collected by vacuum filtration and dried overnight under vacuum (971 mg, 542 µmol, 83% yield). X-ray quality colorless plates were formed by dissolving the precipitate in a minimum amount of hot aceto­nitrile and setting aside for slow evaporation. M.p. 438 K (dec). ^1^H NMR (saturated, CD_3_CN): 8.514 (*d*, 1H, *J* = 5.0), 7.802 (*ddd*, 1H, *J =* 1.7, 7.6, 7.6), 7.373 (*dd*, 1H, *J* = 5.0, 7.9), 4.144 (*d*, 2H, *J* = 4.1), 2.928 (*bm*, 1H), 2.804 (*m*, 1H) 2.668 (*m*, 1H). Analysis calculated for C_32_H_44_Br_4_Hg_4_N_8_S_4_: C, 21.46; H, 2.48; N, 6.26. Found: C, 21.35; H2.45; N, 6.12.

## 7. Refinement

Crystal data, data collection and structure refinement details are summarized in Table 4 ▸. The hydrogen atoms were placed in calculated positions with C—H distances of 0.95 (aromatic) and 0.99 Å (methyl­ene) and refined as riding atoms with *U*_iso_(H) = 1.2*U*_eq_(C).Å

## Supplementary Material

Crystal structure: contains datablock(s) I. DOI: 10.1107/S205698902300823X/yz2039sup1.cif

Supporting information file. DOI: 10.1107/S205698902300823X/yz2039Isup3.cdx

Structure factors: contains datablock(s) I. DOI: 10.1107/S205698902300823X/yz2039Isup4.hkl

CCDC reference: 2296007

Additional supporting information:  crystallographic information; 3D view; checkCIF report

## Figures and Tables

**Table 1:** Selected geometric parameters (Å,°) for **1**

Hg–N2	2.372 (3)	Hg2–S1	2.4802 (9)
Hg1–N4	2.500 (3)	Hg2–S2^i^	2.4826 (9)
Hg1–S1	2.4646 (9)	Hg2–Br1	2.6323 (4)
Hg1–S2	2.4348 (9)	Hg2–Br2	2.7158 (4)
			
N2–Hg1–N4	112.59 (10)	S1–Hg2–S2^i^	127.94 (3)
N2–Hg1–S2	115.39 (8)	S1–Hg2–Br1	108.19 (2)
N4–Hg1–S1	104.57 (7)	S1–Hg2–Br2	101.76 (2)
N4–Hg1–S2	82.25 (7)	Br1–Hg2–S2^1^	105.56 (2)
S1–Hg1–S2	156.40 (3)	Br2–Hg2–S2^i^	104.21 (2)
Hg1–S1–Hg2	96.99 (3)	Br1–Hg2–Br2	107.804 (12)
Hg1–S2–Hg2^i^	97.38 (3)		

Symmetry code: (i) –*x* + 1, –*y* + 1, –*z* + 1

**Table 2:** Overview of pyrid­yl–pyridyl ring geometry metrics (Å,°) for **1** *Cg*1 and *Cg*2 are the centroids of the N1/C1–C5 and N3/C9–C13 rings, respectively.

Centroids	Dihedral angle between rings	Centroid–centroid distance	Centroid–plane distance	Centroid offset
*Cg*1⋯*Cg*2^i^	62.87 (13)	4.873 (2)	4.735 (3)	1.151
*Cg*1⋯*Cg*2^ii^	62.87 (13)	4.453 (2)	4.312 (3)	1.112

Symmetry codes: (i) *x*, *y* − 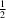 , −*z* + 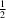 ; (ii) *x*, − *y* + 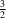 , *z* + 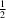 .

**Table 3:** Hydrogen-bond geometry (Å, °)

*D*—H⋯*A*	*D*—H	H⋯*A*	*D*⋯*A*	*D*—H⋯*A*
N4—H4*N*⋯Br2^ii^	1.00	2.95	3.572 (3)	121
C6—H6*A*⋯Br1^iii^	0.99	3.02	3.944 (4)	157
C7—H7*A*⋯Br1^iii^	0.99	3.02	3.978 (4)	163
C15—H15*A*⋯Br2^ii^	0.99	2.86	3.503 (4)	123
C15—H15*B*⋯Br2^i^	0.99	3.09	3.973 (4)	149

Symmetry codes: (i) 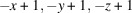 ; (ii) 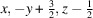 ; (iii) 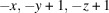 .

**Table 4:** Experimental details

Crystal data	
Chemical formula	[Hg_4_Br_4_(C_8_H_11_N_2_S)_4_]
*M*_r_	1790.99
Crystal system, space group	Monoclinic, *P*2_1_/*c*
Temperature (K)	100
*a*, *b*, *c* (Å)	12.3055 (8), 12.1464 (8), 14.9763 (9)
β (°)	99.509 (1)
*V* (Å^3^)	2207.7 (2)
*Z*	2
Radiation type	Mo *K*α
μ (mm^−1^)	17.71
Crystal size (mm)	0.35 × 0.31 × 0.22

Data collection	
Diffractometer	Bruker *SMART* APEXII CCD
Absorption correction	Numerical (*SADABS*; Krause *et al.*, 2015 ▸)
*T*_min_, *T*_max_	0.024, 0.114
No. of measured, independent and observed [*I* > 2σ(*I*)] reflections	32818, 4414, 4133
*R*_int_	0.027
(sin θ/λ)_max_ (Å^−1^)	0.621

Refinement	
*R*[*F* ^2^ > 2σ(*F* ^2^)], *wR*(*F* ^2^), *S*	0.016, 0.034, 1.18
No. of reflections	4414
No. of parameters	235
H-atom treatment	H-atom parameters constrained
Δρ_max_, Δρ_min_ (e Å^−3^)	0.96, −0.73

Computer programs: *APEX3* (Bruker, 2015 ▸), *SAINT-Plus* (Bruker, 2012 ▸), *SHELXT2014/5* (Sheldrick, 2015*a* ▸), *SHELXL2014/5* (Sheldrick, 2015*b* ▸), *ShelXle* (Hübschle *et al.*, 2011 ▸), *ORTEP-3 for Windows* (Farrugia, 2012 ▸), *Mercury* (Macrae *et al.*, 2020 ▸), *CrystalExplorer21.5* (Spackman *et al.*, 2021 ▸), *OLEX2* (Dolomanov *et al.*, 2009 ▸) and *publCIF* (Westrip, 2010 ▸).

## supplementary crystallographic information

### Crystal data

**Table:**

[Hg_4_Br_4_(C_8_H_11_N_2_S)_4_]	*F*(000) = 1632
*M**_r_* = 1790.99	*D*_x_ = 2.694 Mg m^−^^3^
Monoclinic, *P*2_1_/*c*	Mo *K*α radiation, λ = 0.71073 Å
*a* = 12.3055 (8) Å	Cell parameters from 9997 reflections
*b* = 12.1464 (8) Å	θ = 2.2–26.2°
*c* = 14.9763 (9) Å	µ = 17.71 mm^−^^1^
β = 99.509 (1)°	*T* = 100 K
*V* = 2207.7 (2) Å^3^	Block, colourless
*Z* = 2	0.35 × 0.31 × 0.22 mm

### Data collection

**Table:**

Bruker SMART APEXII CCD diffractometer	4133 reflections with *I* > 2σ(*I*)
Radiation source: fine-focus sealed tube	*R*_int_ = 0.027
φ and ω scans	θ_max_ = 26.2°, θ_min_ = 1.7°
Absorption correction: numerical (SADABS; Krause *et al.*, 2015)	*h* = −15→15
*T*_min_ = 0.024, *T*_max_ = 0.114	*k* = −15→15
32818 measured reflections	*l* = −18→18
4414 independent reflections	

### Refinement

**Table:**

Refinement on *F*^2^	Primary atom site location: other
Least-squares matrix: full	Hydrogen site location: inferred from neighbouring sites
*R*[*F*^2^ > 2σ(*F*^2^)] = 0.016	H-atom parameters constrained
*wR*(*F*^2^) = 0.034	*w* = 1/[σ^2^(*F*_o_^2^) + (0.0066*P*)^2^ + 5.3501*P*] where *P* = (*F*_o_^2^ + 2*F*_c_^2^)/3
*S* = 1.18	(Δ/σ)_max_ = 0.003
4414 reflections	Δρ_max_ = 0.96 e Å^−^^3^
235 parameters	Δρ_min_ = −0.73 e Å^−^^3^
0 restraints	

### Special details

**Table:**

Geometry. All esds (except the esd in the dihedral angle between two l.s. planes) are estimated using the full covariance matrix. The cell esds are taken into account individually in the estimation of esds in distances, angles and torsion angles; correlations between esds in cell parameters are only used when they are defined by crystal symmetry. An approximate (isotropic) treatment of cell esds is used for estimating esds involving l.s. planes.

### Fractional atomic coordinates and isotropic or equivalent isotropic displacement parameters (Å^2^)

**Table:**

	*x*	*y*	*z*	*U*_iso_*/*U*_eq_	
Hg1	0.36010 (2)	0.63859 (2)	0.39709 (2)	0.01596 (4)	
Hg2	0.34257 (2)	0.36960 (2)	0.51023 (2)	0.01555 (4)	
Br1	0.17236 (3)	0.28583 (3)	0.57228 (2)	0.01937 (8)	
Br2	0.41406 (3)	0.54687 (3)	0.61405 (2)	0.01797 (8)	
S1	0.27476 (7)	0.45682 (7)	0.36173 (6)	0.01507 (17)	
S2	0.50720 (7)	0.76760 (7)	0.45273 (6)	0.01603 (18)	
N1	0.2436 (3)	0.8986 (3)	0.4380 (2)	0.0259 (7)	
N2	0.1844 (2)	0.6839 (2)	0.4320 (2)	0.0187 (6)	
H2N	0.196666	0.724631	0.490813	0.028*	
N3	0.1801 (3)	0.7126 (2)	0.1325 (2)	0.0197 (7)	
N4	0.3891 (2)	0.7134 (2)	0.2473 (2)	0.0158 (6)	
H4N	0.344787	0.782017	0.232890	0.024*	
C1	0.2971 (4)	0.9946 (3)	0.4380 (3)	0.0297 (9)	
H1	0.341373	1.017980	0.492738	0.036*	
C2	0.2913 (3)	1.0613 (3)	0.3629 (3)	0.0270 (9)	
H2	0.329526	1.129507	0.366415	0.032*	
C3	0.2293 (4)	1.0275 (3)	0.2829 (3)	0.0301 (10)	
H3	0.224540	1.071203	0.229798	0.036*	
C4	0.1736 (3)	0.9277 (3)	0.2815 (3)	0.0256 (9)	
H4	0.129535	0.902190	0.227357	0.031*	
C5	0.1833 (3)	0.8660 (3)	0.3604 (3)	0.0182 (8)	
C6	0.1227 (3)	0.7578 (3)	0.3633 (3)	0.0206 (8)	
H6A	0.048433	0.771744	0.378057	0.025*	
H6B	0.113785	0.722092	0.303082	0.025*	
C7	0.1260 (3)	0.5811 (3)	0.4463 (3)	0.0183 (8)	
H7A	0.047349	0.597985	0.446802	0.022*	
H7B	0.157278	0.550215	0.506197	0.022*	
C8	0.1341 (3)	0.4954 (3)	0.3737 (2)	0.0184 (8)	
H8A	0.096082	0.524139	0.314866	0.022*	
H8B	0.094329	0.428402	0.387721	0.022*	
C9	0.0708 (3)	0.7033 (3)	0.1080 (3)	0.0215 (8)	
H9	0.029095	0.769155	0.096668	0.026*	
C10	0.0147 (3)	0.6047 (3)	0.0980 (3)	0.0231 (8)	
H10	−0.063114	0.602404	0.080596	0.028*	
C11	0.0758 (3)	0.5094 (3)	0.1142 (2)	0.0221 (8)	
H11	0.040555	0.439620	0.107402	0.026*	
C12	0.1888 (3)	0.5162 (3)	0.1405 (2)	0.0192 (8)	
H12	0.232040	0.451499	0.152687	0.023*	
C13	0.2376 (3)	0.6192 (3)	0.1487 (2)	0.0164 (7)	
C14	0.3616 (3)	0.6329 (3)	0.1735 (2)	0.0196 (8)	
H14A	0.395562	0.560988	0.192635	0.024*	
H14B	0.391918	0.658056	0.119720	0.024*	
C15	0.5074 (3)	0.7401 (3)	0.2651 (2)	0.0175 (7)	
H15A	0.529249	0.772789	0.210122	0.021*	
H15B	0.550610	0.671881	0.279782	0.021*	
C16	0.5324 (3)	0.8206 (3)	0.3433 (2)	0.0192 (8)	
H16A	0.486953	0.887453	0.328254	0.023*	
H16B	0.610658	0.842917	0.349405	0.023*	

### Atomic displacement parameters (Å^2^)

**Table:**

	*U*^11^	*U*^22^	*U*^33^	*U*^12^	*U*^13^	*U*^23^
Hg1	0.01171 (7)	0.01497 (7)	0.02108 (8)	−0.00221 (5)	0.00233 (5)	0.00253 (5)
Hg2	0.01402 (7)	0.01313 (7)	0.01822 (7)	−0.00079 (5)	−0.00112 (5)	0.00195 (5)
Br1	0.01458 (16)	0.01840 (18)	0.02510 (19)	−0.00292 (13)	0.00323 (14)	0.00137 (15)
Br2	0.01992 (17)	0.01448 (17)	0.01915 (18)	−0.00256 (14)	0.00219 (14)	−0.00390 (14)
S1	0.0154 (4)	0.0135 (4)	0.0154 (4)	−0.0004 (3)	0.0002 (3)	0.0004 (3)
S2	0.0140 (4)	0.0129 (4)	0.0204 (5)	−0.0002 (3)	0.0008 (3)	−0.0012 (3)
N1	0.0307 (19)	0.0229 (17)	0.0250 (18)	−0.0046 (15)	0.0073 (15)	0.0007 (14)
N2	0.0165 (15)	0.0159 (15)	0.0244 (17)	0.0005 (12)	0.0055 (13)	−0.0004 (13)
N3	0.0197 (16)	0.0178 (16)	0.0211 (16)	0.0022 (13)	0.0016 (13)	−0.0018 (13)
N4	0.0136 (14)	0.0146 (15)	0.0191 (15)	0.0004 (12)	0.0019 (12)	0.0008 (12)
C1	0.035 (2)	0.026 (2)	0.028 (2)	−0.0085 (18)	0.0036 (19)	−0.0003 (18)
C2	0.030 (2)	0.021 (2)	0.033 (2)	−0.0052 (17)	0.0117 (19)	−0.0024 (17)
C3	0.042 (3)	0.023 (2)	0.029 (2)	0.0056 (18)	0.014 (2)	0.0066 (18)
C4	0.034 (2)	0.020 (2)	0.022 (2)	0.0045 (17)	0.0018 (18)	−0.0015 (16)
C5	0.0160 (17)	0.0160 (18)	0.024 (2)	0.0057 (14)	0.0082 (15)	−0.0016 (15)
C6	0.0155 (17)	0.0208 (19)	0.025 (2)	0.0008 (15)	0.0022 (15)	0.0011 (16)
C7	0.0130 (17)	0.0193 (19)	0.0237 (19)	0.0008 (14)	0.0061 (15)	0.0046 (16)
C8	0.0140 (17)	0.0170 (18)	0.0223 (19)	−0.0032 (14)	−0.0029 (15)	0.0044 (15)
C9	0.0198 (19)	0.021 (2)	0.024 (2)	0.0047 (15)	0.0039 (16)	0.0014 (16)
C10	0.0178 (19)	0.031 (2)	0.0194 (19)	−0.0020 (16)	−0.0010 (15)	−0.0008 (17)
C11	0.025 (2)	0.0187 (19)	0.022 (2)	−0.0064 (16)	0.0012 (16)	0.0016 (16)
C12	0.0233 (19)	0.0178 (18)	0.0162 (18)	0.0021 (15)	0.0024 (15)	0.0027 (15)
C13	0.0187 (18)	0.0174 (18)	0.0130 (17)	0.0024 (14)	0.0022 (14)	−0.0004 (14)
C14	0.0195 (18)	0.0191 (19)	0.0198 (19)	0.0025 (15)	0.0021 (15)	−0.0037 (15)
C15	0.0139 (17)	0.0196 (18)	0.0186 (18)	0.0006 (14)	0.0015 (14)	0.0078 (15)
C16	0.0149 (17)	0.0159 (18)	0.025 (2)	−0.0015 (14)	−0.0011 (15)	0.0082 (15)

### Geometric parameters (Å, º)

**Table:**

Hg1—N2	2.372 (3)	C4—C5	1.387 (5)
Hg1—N4	2.500 (3)	C4—H4	0.9500
Hg1—S1	2.4646 (9)	C5—C6	1.515 (5)
Hg1—S2	2.4348 (9)	C6—H6A	0.9900
Hg2—S1	2.4802 (9)	C6—H6B	0.9900
Hg2—S2^i^	2.4826 (9)	C7—C8	1.520 (5)
Hg2—Br1	2.6323 (4)	C7—H7A	0.9900
Hg2—Br2	2.7158 (4)	C7—H7B	0.9900
S1—C8	1.831 (4)	C8—H8A	0.9900
S2—C16	1.835 (4)	C8—H8B	0.9900
N1—C5	1.332 (5)	C9—C10	1.378 (5)
N1—C1	1.338 (5)	C9—H9	0.9500
N2—C7	1.473 (4)	C10—C11	1.380 (5)
N2—C6	1.478 (5)	C10—H10	0.9500
N2—H2N	1.0000	C11—C12	1.384 (5)
N3—C13	1.338 (5)	C11—H11	0.9500
N3—C9	1.340 (5)	C12—C13	1.384 (5)
N4—C15	1.472 (4)	C12—H12	0.9500
N4—C14	1.473 (4)	C13—C14	1.518 (5)
N4—H4N	1.0000	C14—H14A	0.9900
C1—C2	1.379 (6)	C14—H14B	0.9900
C1—H1	0.9500	C15—C16	1.518 (5)
C2—C3	1.372 (6)	C15—H15A	0.9900
C2—H2	0.9500	C15—H15B	0.9900
C3—C4	1.391 (6)	C16—H16A	0.9900
C3—H3	0.9500	C16—H16B	0.9900
			
N2—Hg1—N4	112.59 (10)	N2—C6—H6B	109.6
N2—Hg1—S2	115.39 (8)	C5—C6—H6B	109.6
N4—Hg1—S1	104.57 (7)	H6A—C6—H6B	108.1
N4—Hg1—S2	82.25 (7)	N2—C7—C8	112.6 (3)
S1—Hg1—S2	156.40 (3)	N2—C7—H7A	109.1
Hg1—S1—Hg2	96.99 (3)	C8—C7—H7A	109.1
Hg1—S2—Hg2^i^	97.38 (3)	N2—C7—H7B	109.1
S1—Hg2—S2^i^	127.94 (3)	C8—C7—H7B	109.1
S1—Hg2—Br1	108.19 (2)	H7A—C7—H7B	107.8
S1—Hg2—Br2	101.76 (2)	C7—C8—S1	114.8 (2)
S2^i^—Hg2—Br1	105.56 (2)	C7—C8—H8A	108.6
S2^i^—Hg2—Br2	104.21 (2)	S1—C8—H8A	108.6
Br1—Hg2—Br2	107.804 (12)	C7—C8—H8B	108.6
C8—S1—Hg1	97.22 (12)	S1—C8—H8B	108.6
C8—S1—Hg2	101.84 (12)	H8A—C8—H8B	107.5
C16—S2—Hg1	98.39 (12)	N3—C9—C10	124.4 (4)
C16—S2—Hg2^i^	101.88 (12)	N3—C9—H9	117.8
C5—N1—C1	117.6 (3)	C10—C9—H9	117.8
C7—N2—C6	114.2 (3)	C9—C10—C11	117.5 (3)
C7—N2—Hg1	108.7 (2)	C9—C10—H10	121.3
C6—N2—Hg1	111.7 (2)	C11—C10—H10	121.3
C7—N2—H2N	107.3	C10—C11—C12	119.5 (3)
C6—N2—H2N	107.3	C10—C11—H11	120.2
Hg1—N2—H2N	107.3	C12—C11—H11	120.2
C13—N3—C9	117.1 (3)	C11—C12—C13	118.7 (3)
C15—N4—C14	112.4 (3)	C11—C12—H12	120.6
C15—N4—Hg1	101.6 (2)	C13—C12—H12	120.6
C14—N4—Hg1	112.5 (2)	N3—C13—C12	122.8 (3)
C15—N4—H4N	110.0	N3—C13—C14	115.6 (3)
C14—N4—H4N	110.0	C12—C13—C14	121.6 (3)
Hg1—N4—H4N	110.0	N4—C14—C13	110.7 (3)
N1—C1—C2	123.5 (4)	N4—C14—H14A	109.5
N1—C1—H1	118.2	C13—C14—H14A	109.5
C2—C1—H1	118.2	N4—C14—H14B	109.5
C3—C2—C1	118.9 (4)	C13—C14—H14B	109.5
C3—C2—H2	120.6	H14A—C14—H14B	108.1
C1—C2—H2	120.6	N4—C15—C16	110.5 (3)
C2—C3—C4	118.4 (4)	N4—C15—H15A	109.5
C2—C3—H3	120.8	C16—C15—H15A	109.5
C4—C3—H3	120.8	N4—C15—H15B	109.5
C5—C4—C3	119.0 (4)	C16—C15—H15B	109.5
C5—C4—H4	120.5	H15A—C15—H15B	108.1
C3—C4—H4	120.5	C15—C16—S2	114.9 (2)
N1—C5—C4	122.6 (4)	C15—C16—H16A	108.6
N1—C5—C6	116.1 (3)	S2—C16—H16A	108.6
C4—C5—C6	121.3 (3)	C15—C16—H16B	108.6
N2—C6—C5	110.4 (3)	S2—C16—H16B	108.6
N2—C6—H6A	109.6	H16A—C16—H16B	107.5
C5—C6—H6A	109.6		
			
C5—N1—C1—C2	1.1 (6)	C13—N3—C9—C10	−0.4 (6)
N1—C1—C2—C3	−1.3 (7)	N3—C9—C10—C11	−0.2 (6)
C1—C2—C3—C4	0.9 (6)	C9—C10—C11—C12	0.8 (6)
C2—C3—C4—C5	−0.5 (6)	C10—C11—C12—C13	−0.8 (5)
C1—N1—C5—C4	−0.6 (6)	C9—N3—C13—C12	0.3 (5)
C1—N1—C5—C6	−179.2 (3)	C9—N3—C13—C14	178.2 (3)
C3—C4—C5—N1	0.3 (6)	C11—C12—C13—N3	0.2 (5)
C3—C4—C5—C6	178.8 (3)	C11—C12—C13—C14	−177.5 (3)
C7—N2—C6—C5	173.9 (3)	C15—N4—C14—C13	−172.3 (3)
Hg1—N2—C6—C5	−62.3 (3)	Hg1—N4—C14—C13	73.7 (3)
N1—C5—C6—N2	−30.2 (4)	N3—C13—C14—N4	51.0 (4)
C4—C5—C6—N2	151.3 (3)	C12—C13—C14—N4	−131.1 (3)
C6—N2—C7—C8	79.5 (4)	C14—N4—C15—C16	−178.4 (3)
Hg1—N2—C7—C8	−45.8 (3)	Hg1—N4—C15—C16	−57.9 (3)
N2—C7—C8—S1	57.4 (4)	N4—C15—C16—S2	64.1 (3)
Hg1—S1—C8—C7	−34.3 (3)	Hg1—S2—C16—C15	−29.0 (3)
Hg2—S1—C8—C7	64.5 (3)	Hg2^i^—S2—C16—C15	70.4 (3)

Symmetry code: (i) −*x*+1, −*y*+1, −*z*+1.

### Hydrogen-bond geometry (Å, º)

**Table:**

*D*—H···*A*	*D*—H	H···*A*	*D*···*A*	*D*—H···*A*
N2—H2*N*···N3^ii^	1.00	2.30	3.264 (4)	163
N4—H4*N*···Br2^iii^	1.00	2.95	3.572 (3)	121
C6—H6*A*···Br1^iv^	0.99	3.02	3.944 (4)	157
C7—H7*A*···Br1^iv^	0.99	3.02	3.978 (4)	163
C15—H15*A*···Br2^iii^	0.99	2.86	3.503 (4)	123
C15—H15*B*···Br2^i^	0.99	3.09	3.973 (4)	149

Symmetry codes: (i) −*x*+1, −*y*+1, −*z*+1; (ii) *x*, −*y*+3/2, *z*+1/2; (iii) *x*, −*y*+3/2, *z*−1/2; (iv) −*x*, −*y*+1, −*z*+1.
